# Crystal structure of (*E*)-undec-2-enoic acid

**DOI:** 10.1107/S2056989015009469

**Published:** 2015-05-28

**Authors:** Marcel Sonneck, Tim Peppel, Anke Spannenberg, Sebastian Wohlrab

**Affiliations:** a Leibniz-Institut für Katalyse e. V. an der Universität Rostock, Albert-Einstein-Strasse 29a, 18059 Rostock, Germany

**Keywords:** crystal structure, hydrogen bond, dimer, unsaturated carb­oxy­lic acid

## Abstract

In the mol­ecule of the title low-melting α,β-unsaturated carb­oxy­lic acid, C_11_H_20_O_2_, the least-squares mean line through the octyl chain forms an angle of 60.10 (13)° with the normal to plane of the acrylic acid fragment (r.m.s. deviation = 0.008 Å). In the crystal, centrosymmetrically related mol­ecules are linked by pairs of O—H⋯O hydrogen bonds into dimers, forming layers parallel to the (041) plane.

## Related literature   

For an adapted direct synthesis of the title compound following the procedure established by Knoevenagel (1898 ▸) and Doebner (1902 ▸), see: Bikulova *et al.* (1988 ▸); Kemme *et al.* (2010 ▸). For crystal structure determinations of related unsat­urated α,β-carb­oxy­lic acids, see, for acrylic acid: Higgs & Sass (1963 ▸); Chatani *et al.* (1963 ▸); Boese *et al.* (1999 ▸); Oswald & Urquhart (2011 ▸); see, for crotonic acid: Shimizu *et al.* (1974 ▸); see, for (*E*)-pent-2-enoic acid: Peppel *et al.* (2015*a* ▸); see, for (*E*)-hex-2-enoic acid: Peppel *et al.* (2015*b* ▸). For structures of co-crystals containing (*E*)-hex-2-enoic acid, see: Aakeröy *et al.* (2003 ▸); Stanton & Bak (2008 ▸).
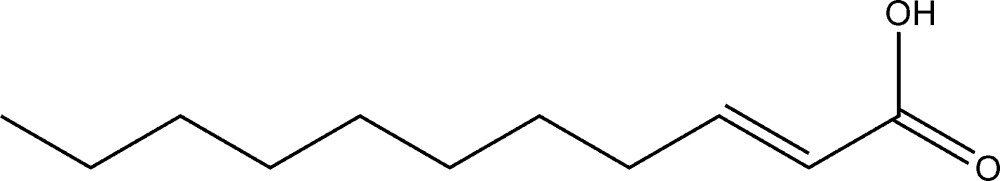


## Experimental   

### Crystal data  

C_11_H_20_O_2_
*M* *_r_* = 184.27
Triclinic, 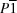

*a* = 4.6346 (4) Å
*b* = 5.4200 (5) Å
*c* = 22.7564 (19) Å
α = 88.386 (2)°
β = 88.357 (2)°
γ = 78.340 (2)°
*V* = 559.46 (8) Å^3^
*Z* = 2
Mo *K*α radiation
μ = 0.07 mm^−1^
*T* = 150 K
0.50 × 0.41 × 0.12 mm

### Data collection  

Bruker APEXII CCD diffractometer
Absorption correction: multi-scan (*SADABS*; Bruker, 2014 ▸) *T*_min_ = 0.87, *T*_max_ = 0.99
13660 measured reflections
2687 independent reflections
2317 reflections with *I* > 2σ(*I*)
*R*_int_ = 0.022

### Refinement  

*R*[*F* ^2^ > 2σ(*F* ^2^)] = 0.047
*wR*(*F* ^2^) = 0.134
*S* = 1.10
2687 reflections
122 parameters
H atoms treated by a mixture of independent and constrained refinement
Δρ_max_ = 0.33 e Å^−3^
Δρ_min_ = −0.24 e Å^−3^

### 

Data collection: *APEX2* (Bruker, 2014 ▸); cell refinement: *SAINT* (Bruker, 2013 ▸); data reduction: *SAINT*; program(s) used to solve structure: *SHELXS97* (Sheldrick, 2008 ▸); program(s) used to refine structure: *SHELXL2014* (Sheldrick, 2015 ▸); molecular graphics: *SHELXL2014*; software used to prepare material for publication: *SHELXL2014*.

## Supplementary Material

Crystal structure: contains datablock(s) I, New_Global_Publ_Block. DOI: 10.1107/S2056989015009469/rz5160sup1.cif

Structure factors: contains datablock(s) I. DOI: 10.1107/S2056989015009469/rz5160Isup2.hkl

Supporting information file. DOI: 10.1107/S2056989015009469/rz5160Isup3.cml

**Fig. 1.**
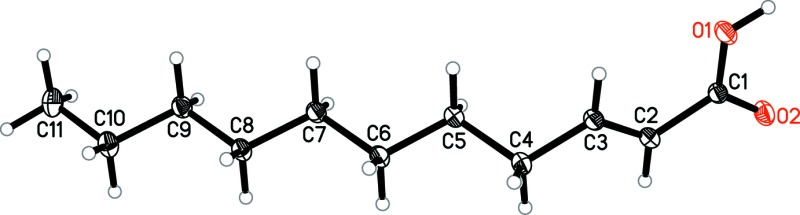
Molecular structure of the title compound with displacement ellipsoids drawn at 30% probability level.

. DOI: 10.1107/S2056989015009469/rz5160fig1.tif
Mol­ecular structure of the title compound with displacement ellipsoids drawn at 30% probability level.

**Fig. 2.**
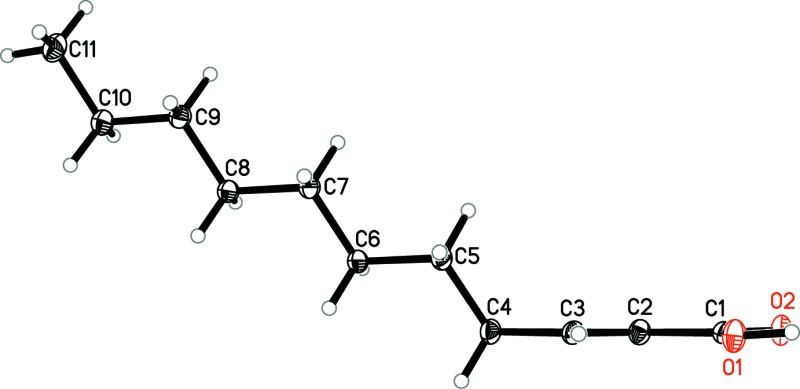
Side view of the molecular structure of the title compound (displacement ellipsoids drawn at 30% probability level).

. DOI: 10.1107/S2056989015009469/rz5160fig2.tif
Side view of the mol­ecular structure of the title compound (displacement ellipsoids drawn at 30% probability level).

**Fig. 3.**
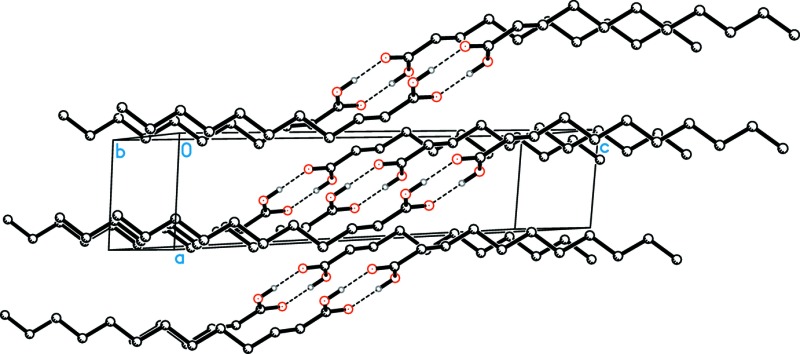
Packing diagram showing intermolecular O—H···O hydrogen bonds. Hydrogen atoms not involved in hydrogen bonding are omitted for clarity.

. DOI: 10.1107/S2056989015009469/rz5160fig3.tif
Packing diagram showing inter­molecular O—H⋯O hydrogen bonds. Hydrogen atoms not involved in hydrogen bonding are omitted for clarity.

CCDC reference: 1401589

Additional supporting information:  crystallographic information; 3D view; checkCIF report

## Figures and Tables

**Table 1:** Hydrogen-bond geometry (, )

*D*H*A*	*D*H	H*A*	*D* *A*	*D*H*A*
O1H1O2^i^	0.90(2)	1.73(2)	2.6244(14)	172.2(19)

Symmetry code: (i) 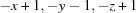.

## supplementary crystallographic information

### S1. Synthesis and crystallization

Malonic acid (25.0g, 240.2 mmol, 1.0 eq) is dissolved in dry pyridine (38.0g, 480.5mmol, 2.0 eq) at room temperature in a three-necked flask equipped with a magnetic stir bar and a reflux condenser under a mild flow of argon. Nonanal (34.2g, 240.2mmol, 1.0 eq) is then added in one portion and the resulting clear solution is further stirred for 72h at room temperature under argon. Afterwards, the resulting light yellow to orange solution is brought to an acidic pH value by adding phospho­ric acid at 0 °C (42.5wt. %, 138.5 g, 600.6mmol, 2.5 eq). The resulting two layers are extracted three times with 150mL portions of ethyl acetate and reduced to a volume of ca. 150 mL *in vacuo*. To remove impurities from aldol condensation the raw acid is converted into the corresponding sodium salt by addition of an aqueous solution of sodium carbonate (20.4 g, 192.2 mmol, 0.8 eq in 200 mL). After stirring for 30 minutes the water phase is separated and extracted three times with 150 mL portions of ethyl acetate. The water phase is then acidified with concentrated hydro­chloric acid (37.0wt. %, 35.5 g, 360.4 mmol, 1.5 eq), the organic phase is separated and the water phase is again extracted three times with 150mL portions of ethyl acetate. The combined organic phases are dried over Na_2_SO_4_ and evaporated to dryness under diminished pressure. The resulting raw product is further purified by distillation *in vacuo* yielding the product in purity >99% (GC). m.p. 18°C. ^1^H NMR (400MHz, CDCl_3_): δ = 12.24 (br s, 1H, OH); 7.09 (dt, ^3^*J* = 15.6 Hz, ^3^*J* = 7.0 Hz, 1H, -CH-); 5.82 (dt, ^3^*J* = 15.6 Hz, ^4^*J* = 1.6 Hz, 1H, -CH-); 2.26-2.19 (m, 2H, -CH_2_-); 1.50-1.43 (m, 2H, -CH_2_-); 1.33-1.24 (m, 10H, 5x -CH_2_-); 0.91-0.85 (m, 3H, -CH_3_-). ^13^C NMR (100MHz, CDCl_3_): δ = 172.50 (CO); 152.69 (CH); 120.76 (CH); 32.47 (CH_2_); 31.98 (CH_2_); 29.48 (CH_2_), 29.32 (CH_2_), 29.29 (CH_2_); 28.02 (CH_2_); 22.79 (CH_2_); 14.22 (CH_3_). MS (EI, 70eV): *m/z* = 184 (M^+^, 0), 99 (15), 97 (12), 96 (11), 95 (11), 86 (17), 84 (17), 83 (17), 82 (17), 81 (16), 73 (36), 70 (17), 69 (25), 68 (20), 67 (19), 57 (37), 56 (20), 55 (46), 54 (12), 53 (23), 45 (22), 43 (60), 42 (20), 41 (100), 40 (14), 39 (57), 29 (62). HRMS (ESI-TOF/MS): calculated for C_11_H_20_O_2_ ([M—H]^-^) 183.13905, found 183.13912. Elemental analysis for C_11_H_20_O_2_ % (calc.): C 71.67 (71.70); H 10.83 (10.94). Suitable single crystals were grown by slow evaporation of an ethano­lic solution at -30 °C over one week.

### S2. Refinement

H1 could be found from the difference Fourier map and was refined with *U*_iso_(H) fixed at 1.5 *U*_eq_(O) . All other H atoms were placed in idealized positions with d(C—H) = 0.95 Å (CH), 0.99 Å (CH_2_), 0.98 Å (CH_3_) and refined using a riding model with *U*_iso_(H) fixed at 1.2 *U*_eq_(C) for CH and CH_2_ and 1.5 *U*_eq_(C) for CH_3_.

### S3. Comment

The crystal structure of (*E*)-undec-2-enoic acid, C_11_H_20_O_2_, an α,β-unsaturated carboxylic acid with a melting point near room temperature (m. p. 18°C), is characterized by acid dimers. The corresponding dimers are connected *via* intermolecular hydrogen bonds of the carboxylic groups C=O···H–O. The crystal packing of (*E*)-undec-2-enoic acid is described by layers of acid dimers parallel to the (0 4 1) plane which are featured by layers of polar headgroups and hydrophobic hydrocarbon chains. The carboxylic group and the following three carbon atoms (C2, C3, C4) of the (*E*)-undec-2-enoic acid molecule lie in one plane (r.m.s. deviation = 0.008 Å), whereas the atoms of the hydrocarbon chain starting from C4 until C11 adopt a nearly fully staggered conformation.

### Crystal data

**Table:**

C_11_H_20_O_2_	*Z* = 2
*M**_r_* = 184.27	*F*(000) = 204
Triclinic, *P*1	*D*_x_ = 1.094 Mg m^−^^3^
*a* = 4.6346 (4) Å	Mo *K*α radiation, λ = 0.71073 Å
*b* = 5.4200 (5) Å	Cell parameters from 7014 reflections
*c* = 22.7564 (19) Å	θ = 2.7–29.0°
α = 88.386 (2)°	µ = 0.07 mm^−^^1^
β = 88.357 (2)°	*T* = 150 K
γ = 78.340 (2)°	Plate, colourless
*V* = 559.46 (8) Å^3^	0.50 × 0.41 × 0.12 mm

### Data collection

**Table:**

Bruker APEXII CCD diffractometer	2687 independent reflections
Radiation source: fine-focus sealed tube	2317 reflections with *I* > 2σ(*I*)
Detector resolution: 8.3333 pixels mm^-1^	*R*_int_ = 0.022
φ and ω scans	θ_max_ = 28.0°, θ_min_ = 1.8°
Absorption correction: multi-scan (*SADABS*; Bruker, 2014)	*h* = −6→6
*T*_min_ = 0.87, *T*_max_ = 0.99	*k* = −7→7
13660 measured reflections	*l* = −30→30

### Refinement

**Table:**

Refinement on *F*^2^	0 restraints
Least-squares matrix: full	Hydrogen site location: mixed
*R*[*F*^2^ > 2σ(*F*^2^)] = 0.047	H atoms treated by a mixture of independent and constrained refinement
*wR*(*F*^2^) = 0.134	*w* = 1/[σ^2^(*F*_o_^2^) + (0.0544*P*)^2^ + 0.245*P*] where *P* = (*F*_o_^2^ + 2*F*_c_^2^)/3
*S* = 1.10	(Δ/σ)_max_ < 0.001
2687 reflections	Δρ_max_ = 0.33 e Å^−^^3^
122 parameters	Δρ_min_ = −0.24 e Å^−^^3^

### Special details

**Table:**

Geometry. All e.s.d.'s (except the e.s.d. in the dihedral angle between two l.s. planes) are estimated using the full covariance matrix. The cell e.s.d.'s are taken into account individually in the estimation of e.s.d.'s in distances, angles and torsion angles; correlations between e.s.d.'s in cell parameters are only used when they are defined by crystal symmetry. An approximate (isotropic) treatment of cell e.s.d.'s is used for estimating e.s.d.'s involving l.s. planes.

### Fractional atomic coordinates and isotropic or equivalent isotropic displacement parameters (Å^2^)

**Table:**

	*x*	*y*	*z*	*U*_iso_*/*U*_eq_	
C1	0.7148 (3)	−0.2848 (2)	0.45696 (6)	0.0231 (3)	
C2	0.8753 (3)	−0.1147 (2)	0.42422 (6)	0.0264 (3)	
H2	0.9723	−0.0090	0.4457	0.032*	
C3	0.8904 (3)	−0.1026 (2)	0.36643 (6)	0.0251 (3)	
H3	0.7934	−0.2107	0.3457	0.030*	
C4	1.0477 (3)	0.0674 (3)	0.33084 (6)	0.0278 (3)	
H4A	1.2051	−0.0358	0.3066	0.033*	
H4B	1.1418	0.1667	0.3576	0.033*	
C5	0.8388 (3)	0.2471 (2)	0.29077 (6)	0.0247 (3)	
H5A	0.7283	0.1481	0.2672	0.030*	
H5B	0.6942	0.3622	0.3154	0.030*	
C6	0.9983 (3)	0.4033 (2)	0.24928 (6)	0.0248 (3)	
H6A	1.1150	0.4972	0.2728	0.030*	
H6B	1.1374	0.2884	0.2236	0.030*	
C7	0.7898 (3)	0.5893 (2)	0.21098 (6)	0.0249 (3)	
H7A	0.6536	0.7060	0.2367	0.030*	
H7B	0.6700	0.4953	0.1883	0.030*	
C8	0.9460 (3)	0.7429 (2)	0.16835 (6)	0.0263 (3)	
H8A	1.0691	0.8340	0.1910	0.032*	
H8B	1.0792	0.6263	0.1421	0.032*	
C9	0.7376 (3)	0.9326 (2)	0.13091 (6)	0.0267 (3)	
H9A	0.6155	0.8415	0.1080	0.032*	
H9B	0.6036	1.0487	0.1571	0.032*	
C10	0.8949 (3)	1.0861 (3)	0.08881 (6)	0.0329 (3)	
H10A	1.0269	0.9703	0.0622	0.039*	
H10B	1.0190	1.1756	0.1116	0.039*	
C11	0.6854 (4)	1.2777 (3)	0.05206 (7)	0.0392 (4)	
H11A	0.5668	1.1901	0.0282	0.059*	
H11B	0.7991	1.3723	0.0262	0.059*	
H11C	0.5552	1.3943	0.0781	0.059*	
O1	0.5924 (2)	−0.43092 (19)	0.42681 (4)	0.0323 (3)	
O2	0.7045 (2)	−0.28190 (19)	0.51201 (4)	0.0320 (3)	
H1	0.490 (5)	−0.520 (4)	0.4504 (9)	0.048*	

### Atomic displacement parameters (Å^2^)

**Table:**

	*U*^11^	*U*^22^	*U*^33^	*U*^12^	*U*^13^	*U*^23^
C1	0.0225 (6)	0.0211 (6)	0.0259 (6)	−0.0050 (5)	−0.0002 (5)	0.0019 (5)
C2	0.0270 (6)	0.0256 (6)	0.0288 (7)	−0.0111 (5)	−0.0007 (5)	0.0023 (5)
C3	0.0239 (6)	0.0227 (6)	0.0294 (7)	−0.0071 (5)	−0.0009 (5)	0.0031 (5)
C4	0.0263 (7)	0.0299 (7)	0.0282 (7)	−0.0093 (5)	0.0012 (5)	0.0064 (5)
C5	0.0245 (6)	0.0253 (6)	0.0255 (6)	−0.0084 (5)	0.0012 (5)	0.0022 (5)
C6	0.0242 (6)	0.0239 (6)	0.0267 (6)	−0.0065 (5)	0.0029 (5)	0.0030 (5)
C7	0.0243 (6)	0.0238 (6)	0.0269 (6)	−0.0065 (5)	0.0012 (5)	0.0023 (5)
C8	0.0255 (6)	0.0239 (6)	0.0292 (7)	−0.0052 (5)	0.0028 (5)	0.0039 (5)
C9	0.0271 (7)	0.0242 (6)	0.0288 (7)	−0.0058 (5)	0.0003 (5)	0.0030 (5)
C10	0.0353 (8)	0.0307 (7)	0.0318 (7)	−0.0059 (6)	0.0037 (6)	0.0064 (6)
C11	0.0485 (9)	0.0335 (8)	0.0338 (8)	−0.0053 (7)	−0.0004 (7)	0.0094 (6)
O1	0.0394 (6)	0.0321 (5)	0.0302 (5)	−0.0195 (4)	−0.0007 (4)	0.0029 (4)
O2	0.0413 (6)	0.0325 (5)	0.0253 (5)	−0.0157 (4)	0.0023 (4)	0.0032 (4)

### Geometric parameters (Å, º)

**Table:**

C1—O2	1.2527 (16)	C7—C8	1.5219 (17)
C1—O1	1.2862 (16)	C7—H7A	0.9900
C1—C2	1.4717 (17)	C7—H7B	0.9900
C2—C3	1.3153 (19)	C8—C9	1.5207 (18)
C2—H2	0.9500	C8—H8A	0.9900
C3—C4	1.4942 (17)	C8—H8B	0.9900
C3—H3	0.9500	C9—C10	1.5173 (19)
C4—C5	1.5289 (18)	C9—H9A	0.9900
C4—H4A	0.9900	C9—H9B	0.9900
C4—H4B	0.9900	C10—C11	1.520 (2)
C5—C6	1.5239 (17)	C10—H10A	0.9900
C5—H5A	0.9900	C10—H10B	0.9900
C5—H5B	0.9900	C11—H11A	0.9800
C6—C7	1.5215 (17)	C11—H11B	0.9800
C6—H6A	0.9900	C11—H11C	0.9800
C6—H6B	0.9900	O1—H1	0.90 (2)
			
O2—C1—O1	123.41 (12)	C8—C7—H7A	108.8
O2—C1—C2	119.25 (11)	C6—C7—H7B	108.8
O1—C1—C2	117.34 (11)	C8—C7—H7B	108.8
C3—C2—C1	122.85 (12)	H7A—C7—H7B	107.7
C3—C2—H2	118.6	C9—C8—C7	113.76 (11)
C1—C2—H2	118.6	C9—C8—H8A	108.8
C2—C3—C4	125.24 (12)	C7—C8—H8A	108.8
C2—C3—H3	117.4	C9—C8—H8B	108.8
C4—C3—H3	117.4	C7—C8—H8B	108.8
C3—C4—C5	111.88 (11)	H8A—C8—H8B	107.7
C3—C4—H4A	109.2	C10—C9—C8	113.44 (11)
C5—C4—H4A	109.2	C10—C9—H9A	108.9
C3—C4—H4B	109.2	C8—C9—H9A	108.9
C5—C4—H4B	109.2	C10—C9—H9B	108.9
H4A—C4—H4B	107.9	C8—C9—H9B	108.9
C6—C5—C4	112.95 (11)	H9A—C9—H9B	107.7
C6—C5—H5A	109.0	C9—C10—C11	113.21 (13)
C4—C5—H5A	109.0	C9—C10—H10A	108.9
C6—C5—H5B	109.0	C11—C10—H10A	108.9
C4—C5—H5B	109.0	C9—C10—H10B	108.9
H5A—C5—H5B	107.8	C11—C10—H10B	108.9
C7—C6—C5	113.02 (11)	H10A—C10—H10B	107.8
C7—C6—H6A	109.0	C10—C11—H11A	109.5
C5—C6—H6A	109.0	C10—C11—H11B	109.5
C7—C6—H6B	109.0	H11A—C11—H11B	109.5
C5—C6—H6B	109.0	C10—C11—H11C	109.5
H6A—C6—H6B	107.8	H11A—C11—H11C	109.5
C6—C7—C8	113.71 (11)	H11B—C11—H11C	109.5
C6—C7—H7A	108.8	C1—O1—H1	110.8 (13)
			
O2—C1—C2—C3	−178.40 (13)	C4—C5—C6—C7	−177.83 (11)
O1—C1—C2—C3	1.88 (19)	C5—C6—C7—C8	−178.77 (11)
C1—C2—C3—C4	179.50 (12)	C6—C7—C8—C9	−178.82 (11)
C2—C3—C4—C5	−119.96 (15)	C7—C8—C9—C10	179.63 (11)
C3—C4—C5—C6	−173.87 (11)	C8—C9—C10—C11	−179.27 (12)

### Hydrogen-bond geometry (Å, º)

**Table:**

*D*—H···*A*	*D*—H	H···*A*	*D*···*A*	*D*—H···*A*
O1—H1···O2^i^	0.90 (2)	1.73 (2)	2.6244 (14)	172.2 (19)

Symmetry code: (i) −*x*+1, −*y*−1, −*z*+1.
