# Supplementary figures and images for: Crystal structure of (E)-undec-2-enoic acid
Source: Acta Crystallogr E Crystallogr Commun. 2015 May 28;71(Pt 6):o426–7. doi: 10.1107/S2056989015009469 (PMC4459382; doi:10.1107/S2056989015009469)

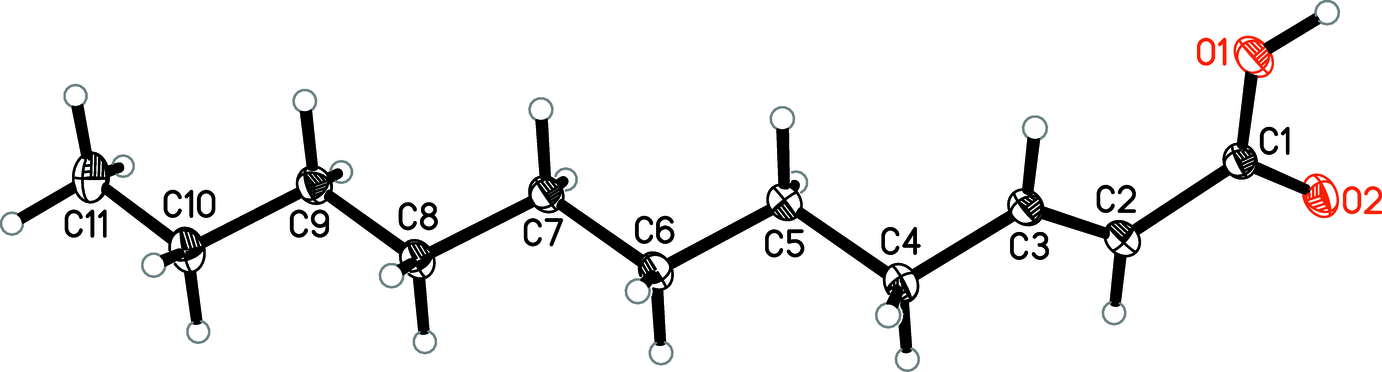

Supplement: Supplementary file 4 [file e-71-0o426-fig1.tif]

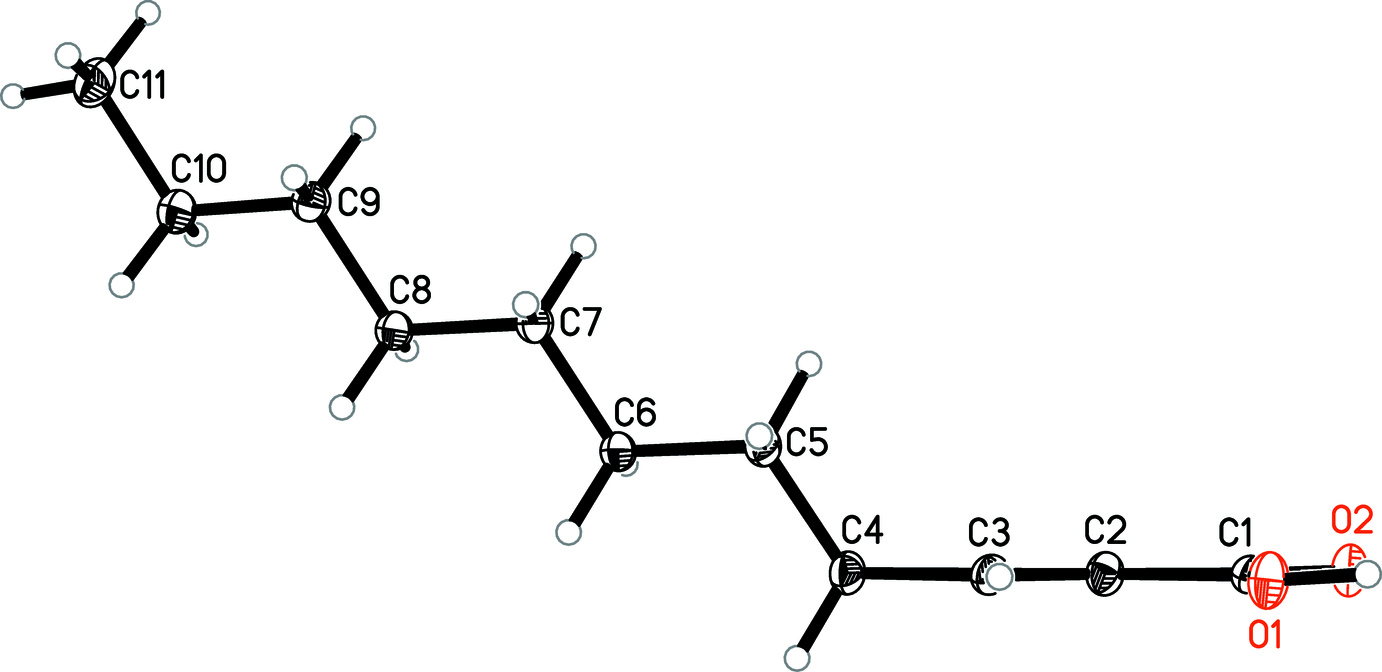

Supplement: Supplementary file 5 [file e-71-0o426-fig2.tif]

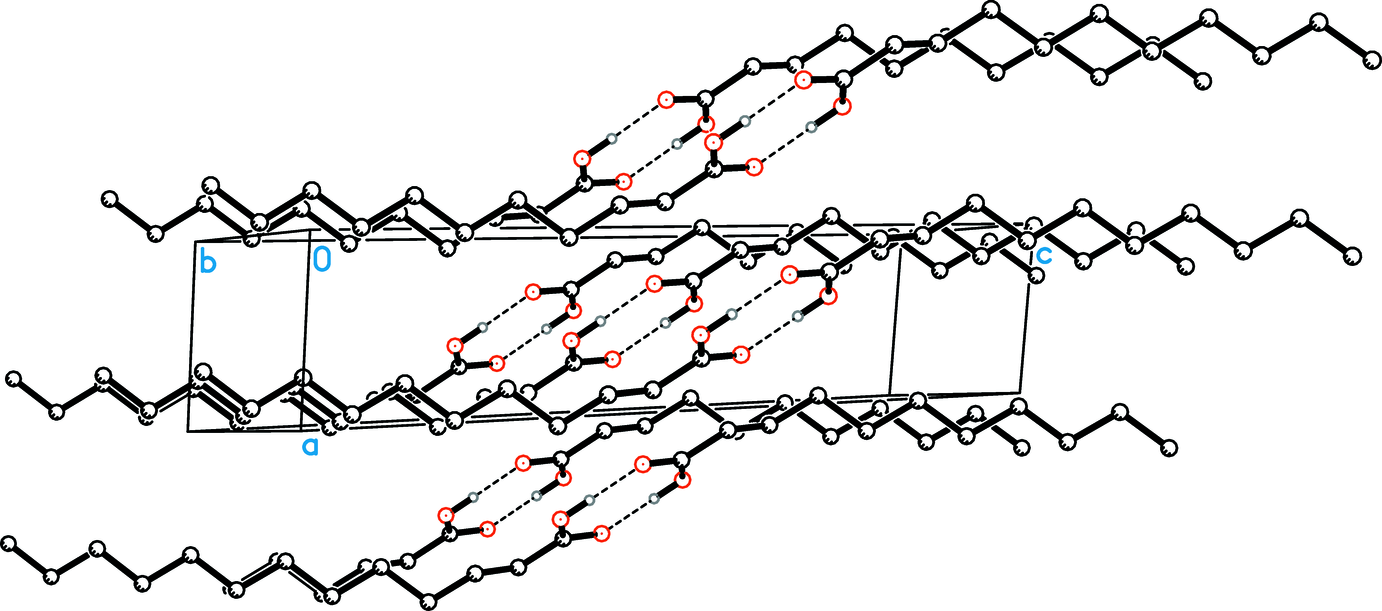

Supplement: Supplementary file 6 [file e-71-0o426-fig3.tif]
